# Associations between Body Composition and Vitamin D Status in Children with Overweight and Obesity Participating in a 1-Year Lifestyle Intervention

**DOI:** 10.3390/nu14153153

**Published:** 2022-07-30

**Authors:** Popi Kasvis, Tamara R. Cohen, Sarah-Ève Loiselle, Tom J. Hazell, Catherine A. Vanstone, Hope A. Weiler

**Affiliations:** 1 Department of Clinical Nutrition, McGill University Health Centre, Montreal, QC H4A 3J1, Canada; popi.kasvis@muhc.mcgill.ca (P.K.); saraheve.loiselle.dtp@gmail.com (S.-È.L.); 2 Faculty of Land and Food Systems| Food, Nutrition and Health, University of British Columbia, Vancouver, BC V6T 1Z4, Canada; 3 Healthy Starts, BC Children’s Hospital Research Institute, Vancouver, BC V5Z 4H4, Canada; 4 Department of Kinesiology & Physical Education, Wilfrid Laurier University, Waterloo, ON N2L 3C5, Canada; thazell@wlu.ca; 5 School of Human Nutrition, McGill University, Montreal, QC H3A 0G4, Canada; catherine.vanstone@muhc.mcgill.ca (C.A.V.); hope.weiler@hc-sc.gc.ca (H.A.W.); 6 Nutrition Research Division, Bureau of Nutritional Sciences, Food Directorate, Health Products and Food Branch, Health Canada, Ottawa, ON K1A 0K9, Canada

**Keywords:** vitamin D, pediatric obesity, anthropometry, body composition, adiposity

## Abstract

Background: To examine associations between body composition and vitamin D status in children participating in a lifestyle intervention. Methods: Children (6–12 y, *n* = 101) with a body mass index (BMI)-for-age >85th percentile were randomized to six dietitian-led behavior counselling sessions or no intervention. Plasma 25-hydroxyvitamin D (25(OH)D), anthropometry, and body composition using dual-energy X-ray absorptiometry were assessed every 3 months for 1 year. For each anthropometry variable (z-scores), tertiles were created to test for differences in 25(OH)D over time (tertile-by-time), and for changes in the z-score (loss, maintain, gain)-by-time, and according to fat patterning (android vs. gynoid) using mixed effects models. Results: The baseline plasma 25(OH)D was 62.2 nmol/L (95%CI: 58.7–65.7), and none < 30 nmol/L. At 6 mo, children with gynoid fat patterning had higher 25(OH)D concentrations than in those with android fat patterning (64.5 ± 1.1 nmol/L vs. 50.4 ± 1.0 nmol/L, *p* < 0.003, Cohen’s *f* = 0.20). Children with the lowest lean mass index z-score at 9 mo had higher plasma 25(OH)D concentrations than children with the highest z-score at baseline, 3 mo, and 6 mo (*p* < 0.05, Cohen’s *f* = 0.20). No other significant differences were observed. Conclusion: In this longitudinal study, vitamin D deficiency was not present in children 6–12 y of age with obesity. Reductions in adiposity did not alter the vitamin D status.

## 1. Introduction

The number of children and adolescents with overweight and obesity has doubled in the last four decades [1]. Canadian Community Health Survey data from 2015 showed that 18.9% of children ages 5–17 y presented with an overweight condition and 12.0% with obesity [2]. The situation is more dire in the United States, with the prevalence of obesity among school-aged children at 18.4% in 2015–2016 [3]. Co-morbidities associated with pediatric obesity, both during childhood and continuing into adulthood, are well known [4,5,6]. However, perhaps counterintuitively, childhood obesity can result in an increased risk of nutrient deficiency. Specifically, a low vitamin D status is common in obesity, and an inverse relationship between body mass index (BMI) and vitamin D status has been observed in both children and adults [7,8,9,10,11].

A sufficient vitamin D status, defined as plasma 25-hydroxyvitamin D (25(OH)D) at or above 50 nmol/L, is not achieved by 30% of children with obesity, despite food fortification in North America [12]. Additionally, a high BMI and excess adiposity may directly affect the bioavailability of the vitamin. It has been postulated [13] that both exogenous and endogenous vitamin D, being a lipophilic molecule, becomes sequestered within adipose tissues [14]. The capacity of fat tissues to sequester vitamin D, and whether a saturation point exists, is unclear. While the mechanistic explanation for sequestration remains elusive, it has been proposed that the volumetric dilution of vitamin D in the larger adipose stores of those with obesity is associated with a failure to achieve vitamin D sufficiency [15,16]. A recent review suggests that both proposed theories could play a role and may partially explain why adults with obesity who undergo Roux-en-Y gastric bypass surgery improved their vitamin D status 6 months postoperatively (from 33.4 ± 22.7 to 56.9 ± 28.2 nmol/L, *p* < 0.0001) [17]. This was coincident with weight loss (30.7 ± 8.1 kg) and while taking only 200–500 IU cholecalciferol daily [18], with variable degrees of adherence to postoperative supplementation [19,20]. Additionally, animal models have demonstrated that energy restriction leads to increased serum 25(OH)D in animals with obesity compared to lean rats, even when the dietary intake is not different and controlled [21].

The information regarding changes in adiposity outcomes and the effect on vitamin D status in children with overweight and obesity is scant [22]. Therefore, the objective of this study was to examine relationships between vitamin D status and body composition in 6–12 y old children with overweight or obesity who participated in a 1-year family-centered lifestyle intervention with the goal of weight reduction.

## 2. Materials and Methods

### 2.1. Design and Subjects

This study is based on secondary use of data collected from January 2011 to February 2014 on 138 participants recruited for the McGill Youth and Lifestyle Intervention with Food and Exercise (MYLIFE) study (NCT01290016); the primary outcome is published [23,24]. School-aged children with overweight and obesity were followed at baseline and then every 3 mo for a period of one year and a total of five visits. The children were randomized into three groups: (1) an intervention group that received individualized counselling based on Canada’s Food Guide for Healthy Eating at the time of the study (2007, CFG group); (2) an intervention group aimed at a subset of children aged 6 to 8.9 y, which was also based on the 2007 food guide, but doubled recommended dairy servings to 4 (milk 4 group); and (3) a control group that did not receive any counselling sessions within the study year. The intervention groups also received advice on increasing physical activity [25], reducing screen time, and recognizing satiety cues. Among those in the intervention groups, all counselling sessions were individualized, including both the children and their families, with the aim of maintaining or reducing body fat within the year [23]. After the year of study visits were complete, the control group received the same counselling sessions as the treatment groups. A flowchart indicating the reasons for exclusion from the analysis is outlined in Supplementary Figure S1.

### 2.2. Inclusion and Exclusion Criteria

#### 2.2.1. Included

Healthy children ages 6.0 to 12.9 y at recruitment, with BMI-for-age >85th percentile according to the World Health Organization (WHO) [26], were included. This analysis included MYLIFE participants who had a complete serial assessment of body composition measurements and blood draws for 25(OH)D.

#### 2.2.2. Excluded

Children with chronic illnesses, such as cancer, Crohn’s disease, nephrotic syndrome, rheumatic conditions, diabetes of any type, or familial hyperlipidemia; bone diseases, such as rickets or osteomalacia, as well as diseases that hinder absorption and metabolism of vitamin D and/or minerals, liver disease, renal disease, current fractures, and abnormalities in parathyroid function; use of medication(s) that affect bone, such as glucocorticoids, phosphate therapy, vitamin D analogues, and bisphophosphates, were all excluded. Severely anemic children were also excluded [27]. This analysis excluded MYLIFE participants who did not have a complete serial assessment of body composition measurements and blood draws for 25(OH)D.

### 2.3. Biochemistry

At each visit, a blood sample was drawn. Participants were asked to fast for 12 h prior to the blood draw, although a small amount of water for hygiene and hydration purposes was permitted. A venous blood puncture was the preferred method for the blood draw; however, capillary punctures were performed on some children and recorded. All blood draws were performed between 0800 and 1200 h. Plasma 25(OH)D concentrations were assessed using a chemiluminescence immunoassay (Liaison, DiaSorin, Saluggia, Italy). The intra-assay percent coefficient of variation for the low 25(OH)D control was 2.0% (39.6 ± 0.3 nmol/L), whereas the high 25(OH)D control was 1.9% (113.1 ± 2.3 nmol/L). The inter-assay percent coefficient of variation between the low and high controls was 2.0%. Quality control measures of this assay method included participation in Vitamin D External Quality Assurance Scheme (DEQAS) and use of National Institute of Standards and Technology (NIST) guidelines. The inter-assay percent coefficient of variation in the NIST samples was 2.6% (74.3 ± 1.6 nmol/L) for the high control and 4.0% (46.1 ± 1.1 nmol/L) for the low control. Accuracy for the high and low controls was within 5% of certified values. The National Academy of Medicine (formally known as the Institute of Medicine (IOM)) cut-offs based on 25(OH)D concentrations for bone health were used to establish vitamin D status as follows: sufficiency at ≥50 nmol/L, risk of inadequacy at 30–50 nmol/L, and risk of deficiency symptoms at <30 nmol/L [28].

### 2.4. Anthropometry

At each visit, weight, height, and waist circumference were taken and BMI was calculated using standardized methods previously reported [27]. BMI z-scores were calculated using the WHO AnthroPlus software (AnthroPlus, Geneva, Switzerland, 2009). Waist-to-height ratio (waist:height) was calculated: WC (cm)/height (cm) [29]. Body composition was measured at every visit using dual-energy X-ray absorptiometry (DXA) (Hologic Inc., Bedford, MA, USA). Daily calibration of the DXA was performed using a phantom spine (Hologic phantom #14774) provided by the manufacturer; additionally, radiographic uniformity tests were maintained within established limits across the study. Measures of whole-body fat mass (FM; kg), percent body fat (%BF), and lean body mass (LBM; kg), excluding bone mineral content, were determined using Apex software version 13.3:3. From these measures, FM index (FMI) was calculated in the following manner: FM (kg)/[height (m)]^2^. Similarly, lean mass index (LMI) was calculated as follows: LBM (kg)/[height (m)]^2^. FMI and LMI calculations based on height that were raised to the power of 2.5 and 3, respectively, as proposed by Ofenheimer et al. [30], were explored. As these exponents did not render significant results, FMI and LMI calculated with height squared are reported. Regional analysis of trunk and appendicular FM and android–gynoid ratio (android:gynoid), as determined by the Apex software, was recorded. Android fat analysis performed in this study was defined as the sum of all adipose tissue (subcutaneous and visceral) between the anterior pelvis and the mid-point of the lumbar spine. Adipose tissue located between the femur head and mid-thigh (subcutaneous or intramuscular) was considered the gynoid region. Trunk:limb FM ratio was calculated as: trunk FM (kg)/[arm FM (kg) + leg FM (kg)] [30]. Z-scores were generated for WC, waist:height [29], %BF, FMI, LMI, and trunk:limb FM [30] using published lambda (skew), mu (median), and sigma (generalized coefficient of variation) (LMS) parameters with the following equation: z = (measure/M)^L^−1/(L/S) [31]. In each case, the baseline measure was used to create the tertiles based on LMS parameter for age and sex. Each adiposity indicator z-score was then separated into tertiles to allow for testing of potential differences in plasma 25(OH)D concentration: tertile 1 contains one third of children with the lowest z-score for each adiposity indicator, tertile 3 contains one third of children with the highest z-score for each adiposity indicator, and tertile 2 contains children that fall between these two points. The children within each indicator tertile group fluctuated, since individuals potentially fell into a different tertile for each adiposity indicator. For example, only 21% of children were classified as tertile 1 across all adiposity indicators, 3% in tertile 2, and 26% in tertile 3; the other 50% were categorized in contiguous or opposite tertiles. As no LMS data are available for android:gynoid, children were categorized as having either android fat patterning (android:gynoid ≥ 1) or gynoid fat patterning (android:gynoid < 1). Finally, participants were categorized as either having lost (Δ z-score < −0.5), maintained (Δ z-score −0.5–0), or gained (Δ z-score > 0) for each body composition z-score at the 12 mo visit compared to baseline.

### 2.5. Assessment of Vitamin D Sources

Skin pigmentation was measured at each time point using a hand-held spectrophotometer (Konica Minolta, Ramsey, NJ, USA) to determine endogenous vitamin D synthesizing capacity; three measurements were taken at the forehead, forearm, and lateral lower leg to assess facultative pigmentation and the inner upper arm to determine constitutive (natural) pigmentation. Skin color was determined using the Commission international de l’éclairage criteria, where L* values indicate brightness and b* values the blue-yellow [32]. Individual topology values (ITA°) were then calculated using the following formula: ITA° = [arctan((L − 50)/b)] × 180/π. The values from the inner upper arm were used to categorize natural skin color according to the Fitzpatrick scale [33]: light skinned (Fitzpatrick I-III) or dark skinned (Fitzpatrick IV–VI).

A questionnaire to assess children’s sun exposure was adapted from the 2008 Canadian Community Health Survey [34] and completed by parents at each visit. Responses to questions regarding time spent outdoors, the amount of skin exposed to the sun, visits to southern destinations, and sunscreen use allowed for comparison of spectrophotometry readings to reported UVB exposure and calculation of a sun index. The sun index was determined in the following manner: time spent exposed to UV light (hours)*percent of body surface area exposed to the light (%). Body surface area was calculated using Lund–Browder classification [35]; in making this calculation, only one half (one side) of the body exposed to the sun was considered. Season of the year, as it relates to endogenous vitamin D synthesis by UVB exposure (low UVB period = October through March, high UVB period = April through September) [36], was considered in the assessment of vitamin D status.

Exogenous vitamin D intake was estimated at each visit through three 3-day food diaries (analysis performed with Nutritionist Pro, Axxya Systems, Stafford, TX, USA, including Canadian Nutrient File 2010b). Daily vitamin D content was assessed and averaged over the three-day period reported in each diary. Food diaries were completed for two non-consecutive weekdays and one weekend day. Parents were asked about their child’s supplement use at every visit; children were then categorized as supplement users or non-supplement users. Dietary intake of vitamin D is expressed as IU/d.

### 2.6. Pubertal Status

Tanner pubertal stage was reported by the parents at baseline, with children categorized into two categories: (1) ≤stage 3, (2) stages 4 and 5. Tanner stage was not assessed at any other time point.

### 2.7. Socio-Economic/Demographic Assessment

A questionnaire was used at baseline to assess socioeconomic status and demographic information. Ethnicity was self-reported and categorized as White, Black, Hispanic, Asian (includes East Asian, Southeast Asian, Southern Asian), other, or mixed in children with parents of different ethnicities. Questions regarding household family income and maternal education were based on the Canadian Community Health Survey (CCHS), cycle 2.2 [37]. Each child was then categorized as being above or below the median household income for the study group (CAD 75,000/year). Children were also categorized as having mothers with education above or below the university level.

### 2.8. Statistical Analysis

Mixed effects models were used to determine changes over time in plasma 25(OH)D according to the following fixed effects and their interactions: (1) visit (baseline, 12 mo) by loss, gain, or maintenance of z-score for each adiposity indicator and (2) visit (baseline, 3 mo, 6 mo, 9 mo, 12 mo) by z-score tertile. Covariates in these models included UVB exposure period, constitutive skin pigmentation, sunscreen use, and dietary vitamin D intake; sex and age were included in models examining interactions with android:gynoid, since z-scores could not be calculated. The Shapiro–Wilk test demonstrated non-Gaussian distribution of the residuals for these models; therefore, a log transformation was performed on the 25(OH)D variable, and back transformed for the purposes of reporting the results. Covariance structures were tested, with best fit based on the Bayesian information criterion (BIC). Similarly, mixed effects models were used to determine the interaction of visit (baseline, 3 mo, 6 mo, 9 mo, 12 mo) by UVB exposure period on plasma 25(OH)D. This model controlled for age, sex, dietary vitamin D intake, constitutive skin color, and sunscreen use. Treatment group, Tanner stage, supplement use, visits to destinations <40° N latitude during the non-UVB exposure period, venous versus capillary blood draw, mother’s education, and family income were explored in all models, but did not improve the fit based on BIC, and thus were not included in the final models. A final mixed effects model was performed to determine the change in adiposity indicator z-score by loss, gain, or maintenance of z-score for each adiposity indicator and visit (baseline, 12 mo). For all mixed models, reported *p*-values were calculated using Tukey–Kramer post hoc adjustment. Effect sizes for the interactions of each mixed model were calculated and expressed as Cohen’s *f*, where 0.1 is deemed as small, 0.25 as medium, and 0.4 as a large effect [38]. Descriptive characteristics at baseline were explored for sex differences using Student’s *t*-test or the Wilcoxon–Mann–Whitney-U test, in cases of non-Gaussian distributions; estimated effect sizes were reported as Cohen’s *d*, where 0.2 is deemed as small, 0.5 as medium, and 0.8 as a large effect [39].

## 3. Results

### 3.1. Baseline Characteristics

A total of 101 children (males: *n* = 46, females: *n* = 55) were included in this analysis (Supplementary Figure S1). The mean 25(OH)D concentration at the baseline for the whole cohort was 62.2 ± 17.7 nmol/L (95%CI: 58.7–65.7 nmol/L), with 70% of children meeting the IOM 50 nmol/L target in support of bone health in individuals; using the population target, 91% were ≥40 nmol/L, 9% were between 30 and 40 nmol/L, and no children exhibited deficiency (<30 nmol/L) (Table 1). Significant differences between sexes were found at the baseline: males had a greater BMI z-score (Cohen’s *d* = 0.07), WC (Cohen’s *d* = 0.44), waist:height (Cohen’s *d* = 0.52), LBM (Cohen’s *d* = 0.34), LMI (Cohen’s *d* = 0.57), adjusted LMI (Cohen’s *d* = 0.63), and sun index (Cohen’s *d* = 0.25), whereas females had a greater %BF (Cohen’s *d* = 0.46) (Table 1). The sun index was greater in males likely due to a significantly greater proportion of males recruited during the high UVB exposure period (χ^2^ = 0.04). Most children were White, had a Tanner stage <3, were classified as having obesity, had mothers who had completed university, and had a family income that was greater than CAD 75,000 (Table 1). At the baseline, only one child met the recommended dietary allowance (RDA) of 600 IU/d of vitamin D intake through diet, whereas four children met the estimated average requirement (EAR) of 400 IU/day (Table 1).

### 3.2. Adiposity Indicators by Tertile and Change in 25(OH)D over Time

**Figure 1:**
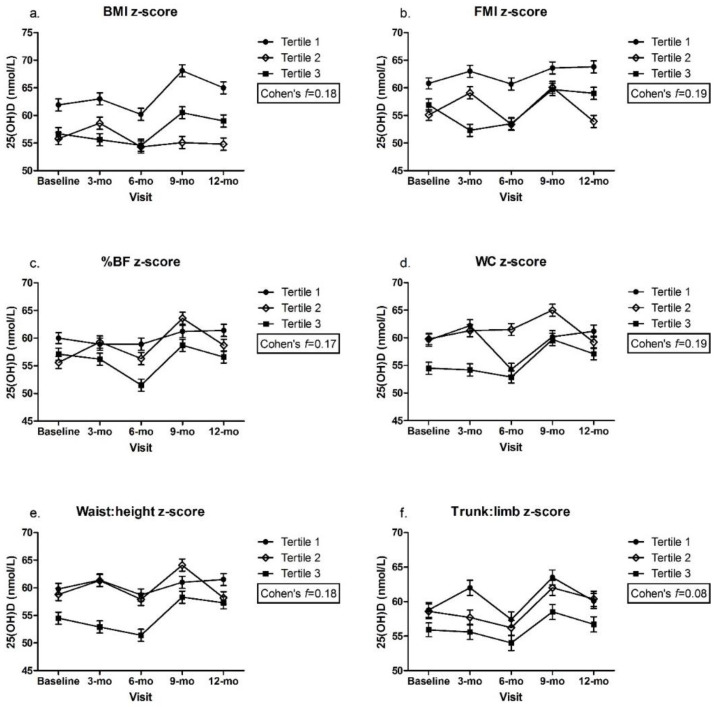
(**a**). BMI z-score; (**b**). FMI z-score; (**c**). %BF z-score; (**d**). WC z-score; (**e**). Waist:height z-score; (**f**). Trunk:limb z-score. Plasma 25(OH)D across adiposity indicator (z-scores) by visit. Mixed effects model, mean ± SEM. Covariates include UVB exposure period, constitutive skin color, sunscreen use, dietary vitamin D intake. Values with same superscripts are significantly different from each other; *p* < 0.05, *n* = 101. Effect size reported as Cohen’s *f*. Abbreviations: 25(OH)D = 25-hydroxyvitamin D; Android:gynoid = android–gynoid ratio; BF = body fat; BMI = body mass index; FMI = fat mass index; height:weight = height-to-weight ratio; LMI = lean mass index; trunk:limb = trunk-to-limb ratio; waist:height = waist-to-height ratio; WC = waist circumference.

**Figure 2:**
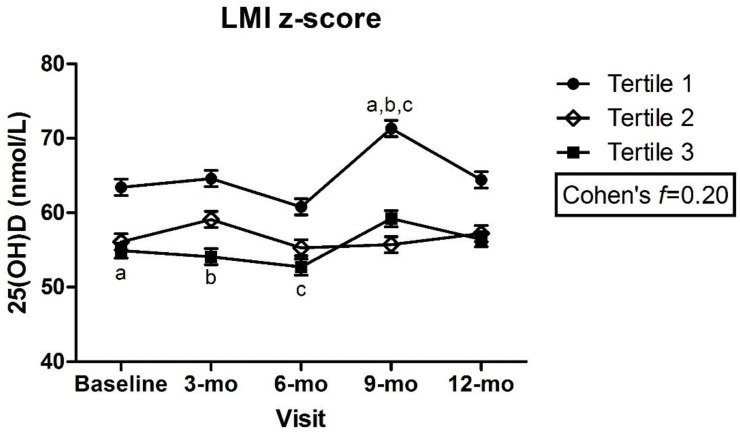
Plasma 25(OH)D across lean mass index z-scores by visit. Mixed effects model, mean ± SEM. Covariates include UVB exposure period, constitutive skin color, sunscreen use, dietary vitamin D intake. Values with same superscripts are significantly different from each other; *p* < 0.05, *n* = 101. Effect size reported as Cohen’s *f*. Abbreviations: 25(OH)D = 25-hydroxyvitamin D; LMI = lean mass index.

There were no significant differences in plasma 25(OH)D within or between z-score tertiles over time for any of the adiposity indicators, with a small to medium effect size for all (Figure 1a–e), except trunk:limb, for which, a medium to large effect size was estimated (Figure 1f). There was a significant difference in plasma 25(OH)D between children in the lowest LMI z-scores (tertile 1) at the 9 mo visit and those with highest LMI z-scores (tertile 3) at baseline, 3 mo, and 6 mo (tertile 1: 9-mo = 71.3 ± 1.1 vs. tertile 3: baseline = 54.9 ± 1.0, 3-mo = 54.1 ± 1.1, 6-mo = 52.7 ± 1.1 nmol/L, *p* < 0.05) (Figure 2). Despite this statistical significance, the actual mean difference was only small to medium (Cohen’s *f* = 0.20) (Figure 2). The mean 25(OH)D remained above the 50 nmol/L cut-point among all z-score tertiles (1, 2, and 3) for all adiposity indicators and at all time points.

**Figure 3:**
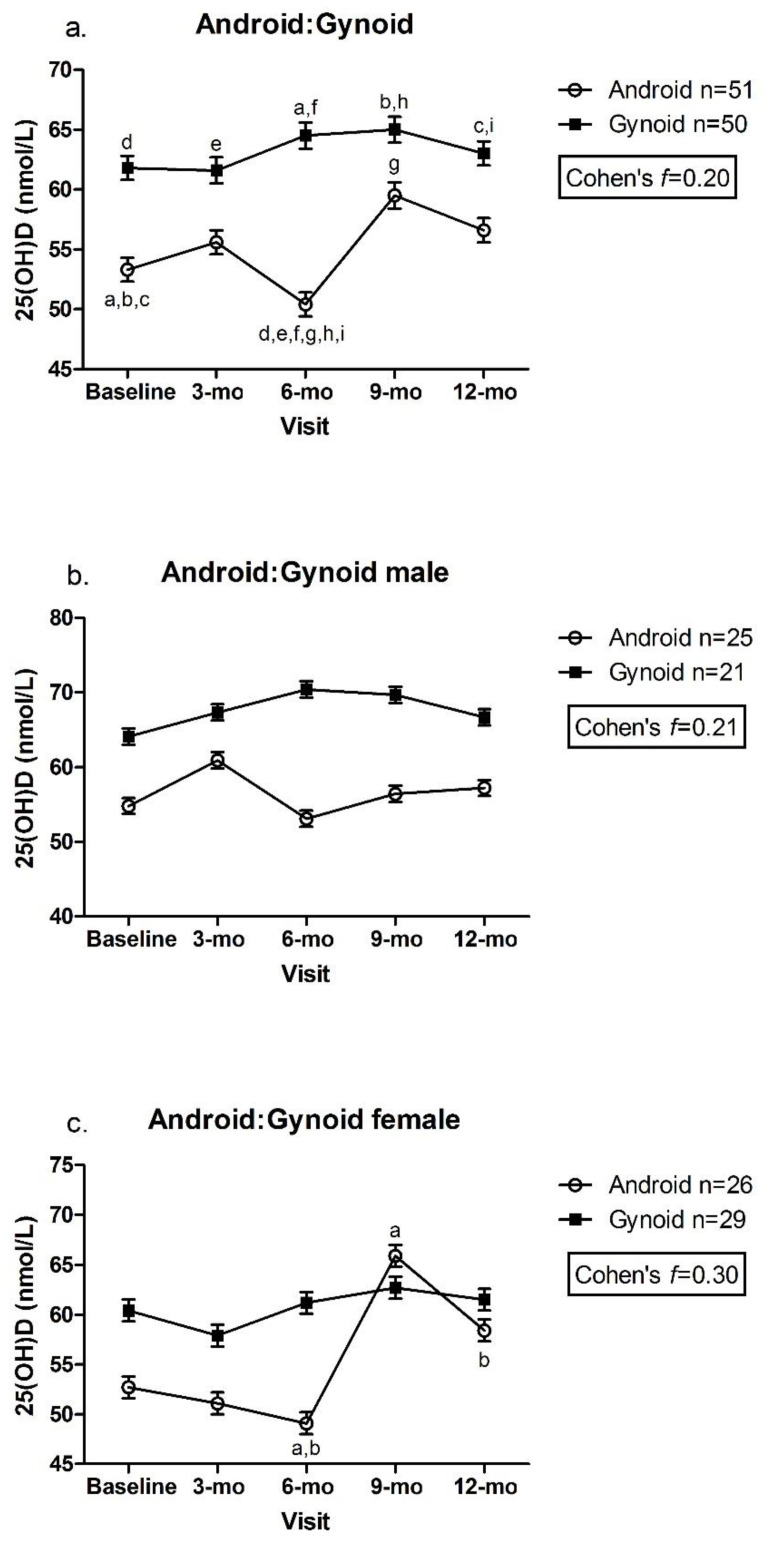
(**a**). Android:Gynoid; (**b**). Android:Gynoid male; (**c**). Android:Gynoid female. Plasma 25(OH)D across android:gynoid fat patterning by visit. Mixed effects model, mean ± SEM. Covariates include UVB exposure period, constitutive skin color, sunscreen use, dietary vitamin D intake. Values with same superscripts are significantly different from each other; *p* < 0.05, *n* = 101. Effect size reported as Cohen’s *f*.

Children classified as having gynoid fat patterning had significantly greater concentrations of 25(OH)D at the 6 mo visit than children with android fat patterning (gynoid: 64.5 ± 1.1 vs. android: 50.4 ± 1.0 nmol/L, *p* < 0.003) (Figure 3a). There was also a significant increase in plasma 25(OH)D concentration between the 6 and 9 mo visit in children with android fat patterning (6 mo: 50.4 ± 1.0 vs. 9 mo: 59.5 ± 1.1 nmol/L, *p* = 0.025) (Figure 3a). There were no between or within-group differences in males with android vs. gynoid fat patterning, with only a small to medium effect size (Cohen’s *f* = 0.21) (Figure 3b). However, females with android fat patterning demonstrated greater plasma 25(OH)D concentrations at the 9 and 12 mo visits (9 mo: 65.9 ± 1.1, 12 mo: 58.4 ± 1.1 nmol/L) compared to the 6 mo visit (6 mo: 49.1 ± 1.1 nmol/L, *p* < 0.05, Cohen’s *f* = 0.30) (Figure 3c).

**Figure 4:**
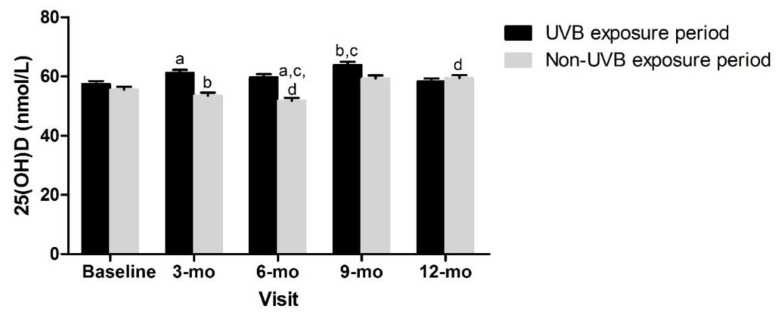
Vitamin D status over time and according to UVB period. Mixed effects model, mean ± SEM. Covariates included sex, age, constitutive skin color, sunscreen use, dietary vitamin D intake. Measures with same superscripts are significantly different from each other; *p* < 0.05.

Given these observed differences, we explored possible explanations beyond the adiposity indicators. No differences between tertiles 1, 2, or 3 for each adiposity indicator z-score or between children with android or gynoid fat patterning were observed at any timepoint for the dietary vitamin D intake (data not shown). Overall, the plasma 25(OH)D was not significantly different between those tested during the UVB exposure period versus those who were not (Figure 4).

### 3.3. Effect of Body Composition Change between Baseline and 12-mo on 25(OH)D Concentrations

There were differences over time in all of the adiposity indicator z-score groups (lost, gained, or maintained) between the baseline and 12 mo visit (*p* ≤ 0.01) (Table 2). For the android:gynoid ratio, there was a significant change in those who lost or gained (*p* < 0.0001), but not in those who maintained. A large effect size for all time *z-score changes was observed (Table 2). Despite this, no significant differences in plasma 25(OH)D were observed in those with decreased, increased, or maintained adiposity measure z-scores between the baseline and 12 mo (Table 3). There was a medium effect on 25(OH)D by z-score change between the baseline and 12 mo in trunk:limb. A low to medium effect on 25(OH)D was observed for the change in BMI z-score, FMI z-score, %BF z-score, WC z-score, waist:height z-score, and LMI z-score (Table 3). There was a low to medium effect on 25(OH)D by the change in android:gynoid (Table 3).

## 4. Discussion

This longitudinal study explored associations between adiposity and plasma 25(OH)D in children with overweight and obesity participating in a 1-year family-centered lifestyle intervention. Children who participated in this study were predominantly vitamin D sufficient, with very few at risk of inadequacy (i.e., <40 mmol/L), as measured by plasma 25(OH)D. Children with the lowest LMI z-scores and with gynoid fat patterning had higher 25(OH)D concentrations than those with the highest LMI z-scores and android fat patterning, respectively. Finally, we observed that a significant loss of adiposity, both whole body and regional, over the year-long intervention did not elicit a significant change in plasma 25(OH)D.

The vitamin D status of participants in this study was consistent with Canadian statistics on school-aged children, with children being classified with a BMI in the overweight and obese ranges [12]. On average, the children in this study met the IOM recommendations for sufficient plasma 25(OH)D (62.2 ± 17.7 nmol/L), despite having overweight/obesity. At baseline, 30% of children in our study did not meet the IOM cut-off of 50 nmol 25(OH)D/L (43.0 nmol/L, 95% CI: 41.1–44.9 nmol/L), but no child exhibited vitamin D deficiency (<30 nmol/L). This is similar to CHMS data, in which, 30% of children aged 6 to 11 y were below IOM targets, and 6% were deficient [12]. This demonstrates that our data are relevant, and likely not biased by inclusion in a trial. The dietary intake does not fully explain the vitamin D status, as both national data and this study demonstrate inadequate intakes. Canadian data in children 9 to 13 y reveal an average intake of 280 IU/day in males and 228 IU/day in females [40]. Our study demonstrated that, even in children with the lowest dietary vitamin D intake (tertile 1: 76.9 ± 35.6 IU/d), the plasma 25(OH)D remained >50 nmol/L (Supplemental Table S2). Additionally, sun index measures in our study demonstrated that children were going outside and were thus likely synthesizing vitamin D during the UVB exposure period; significantly greater sun exposure was seen in males than females, yet males and females were not different in 25(OH)D concentrations. Finally, significantly greater z-scores in some adiposity indicators between sexes did not translate to differences in plasma 25(OH)D; for example, males had higher BMI z-score than females at the baseline, yet there were no differences in 25(OH)D concentrations.

The surprising finding in this study was higher 25(OH)D concentrations in children with the lowest z-score for LMI compared to the highest LMI z-score. This may be explained by other observations, such as those with the most LBM also have higher FM that is prone to sequester vitamin D [41,42]. None of the practical, indirect measures of adiposity commonly used in the clinical arena demonstrated any significant differences in vitamin D status (e.g., BMI, WC, waist:height). This may be partly explained by the homogeneity of the group and the lack of a control group of children within recommended limits for adiposity indicators. On the other hand, the potential negative effect of central adiposity on vitamin D status was demonstrated by the significantly lower plasma 25(OH)D in children presenting with android fat patterning at the 6 mo visit compared to those with gynoid fat patterning. A lower vitamin D status in those with android fat patterning has been recently demonstrated in a large cohort study of Chinese children aged 6–18 y; the android fat mass index in boys was associated with greater odds of vitamin D insufficiency (OR: 1.35 (95% CI:1.20–1.51), *p* < 0.001) [43]. This association was not observed in girls. In adults, a study of Chinese men with overweight and obesity demonstrated that greater visceral fat is an independent factor in the inverse relationship between 25(OH)D and BMI [44].

No statistically significant changes in 25(OH)D were reported based on loss, maintenance, or gain in z-score for each adiposity indicator from baseline to 12 mo; this is despite significant z-score changes in all of these groups. This may reflect the lack of power due to an inadequate sample size to capture changes in 25(OH)D (e.g., power = 0.44 based on 25(OH)D changes by BMI z-score tertile). In addition, while just over 25% of children reduced their BMI z-score, (*n* = 26), this does not necessarily reflect a loss of adiposity. BMI is not a sensitive measure of adiposity and may be reduced by increases in height rather than a change in FM. Fewer children had z-score reductions in direct measures of adiposity (%BF: *n* = 15, FMI: *n* = 17). Changes in FM may also reflect a proportional change related to growth rather than a direct effect of the lifestyle intervention. As such, these small yet meaningful changes in adiposity did not result in significant changes in 25(OH)D concentration in children. Finally, different cutoffs defining loss, maintenance, and gain were explored; we chose the cutoff of ≥−0.5 z-score as a clinically meaningful indicator of loss [45]. This cutoff was quite stringent, as most children in this study experienced modest changes in body composition, as is expected in growing children.

However, a medium to large effect on 25(OH)D from the change in trunk:limb FM was observed. This may be a result of the mean z-score for each tertile not exceeding one standard deviation of the mean at the baseline (tertile 1 = −0.7 ± 0.4, tertile 2 = 0.1 ± 0.2, tertile 3 = 0.8 ± 0.4; Supplementary Table S2), indicating that trunk:limb FM may not be a useful measure of adiposity change in children with overweight or obesity. We also found a low to medium effect on 25(OH)D from changes in overall adipose loss (e.g., BMI and FMI) and central fat reduction (e.g., WC and waist:height). This may suggest that the location of lost adipose tissue may not be an important indicator of 25(OH)D. To the authors’ knowledge, these are the first reported findings of this nature. Kouda et al. previously reported a sex difference in fat patterning among a cohort of Japanese children [22], where there was an inverse relationship between trunk and appendicular fat and vitamin D status in males, but not females. In a large cross-sectional study of adults, both visceral and subcutaneous abdominal fat were inversely related to 25(OH)D [46]. This observation was also observed in adult men undergoing a 1-year lifestyle intervention; there was an inverse relationship between 25(OH)D and the change in visceral (r = −0.36, *p* < 0.0005) and change in total abdominal (r = −0.37, *p* < 0.0005) adipose tissue volumes [47].

### Strengths and Limitations of This Study

This study is novel in its longitudinal examination of changes in adiposity and associations with plasma 25(OH)D. The year-long study design allowed for the collection of repeated measures of multiple adiposity (both via DXA and clinically accessible measures) and vitamin D indicators, dietary intake, sun exposure/sunscreen use, and skin pigmentation. A key strength of this study includes the ranking of children by the LMS calculated z-score for each adiposity indicator. The use of z-scores is advantageous, as it allows for the classification of children based on reference values for sex and age [48]. The direct and objective measurement of skin pigmentation using a spectrophotometer provides valid information on the variations in color resulting from UV exposure over time. Spectrophotometry has been validated as an accurate and reproducible measures of skin pigmentation [49,50], and is likely more accurate than surveyed sun exposure. Despite not being a factor in our final analysis, collecting spectrophotometry measures identifying facultative pigmentation allowed us to explore its relevance in our cohort.

This study is not without limitations. First, this study was a secondary analysis of a study powered to elicit changes in BMI z-scores, not changes in vitamin D status [23]. Second, we did not include a control group of children with a BMI z-score within the acceptable range; the narrow range of the BMI z-score across tertiles may have undermined the likelihood of finding differences in the vitamin D status. As such, the homogeneity of the group does not allow for a generalization of the conclusions beyond this group of children with overweight and obesity. The cohort was also homogeneous in terms of skin color, family income, and mother’s education level, leading to little opportunity to determine how these variables affected the vitamin D status of the group.

## 5. Conclusions

The present study demonstrated that the vitamin D status of school-aged children with overweight and obesity, and without vitamin D deficiency, is, on average, consistent with IOM recommendations, with the majority maintaining 25(OH)D ≥ 50 nmol/L over the 1-year study period. A significant improvement in plasma 25(OH)D was not observed in the children who lost, compared to those who maintained or gained, in each of the adiposity indicators studied, possibly due to an adequate vitamin D status among the majority of children at the baseline. Future investigations could confirm these observations through adequately powered studies that include normal weight children as controls.

## Figures and Tables

**Table 1:** Baseline characteristics of children included in this study.

	Females *n* = 55			Males *n* = 46				
	Mean ± SEM	Min	Max	Mean ± SEM	Min	Max	*p*	ES
Age (y) ^a^	9.0 ± 0.3	6.0	12.9	9.7 ± 0.3	6.8	12.9	0.111	0.34
Height (cm)	139.3 ± 1.9	111.5	178.9	142.4 ± 1.6	126.0	169.5	0.219	0.25
Height z-score	1.0 ± 0.1	−0.7	3.8	1.1 ± 0.1	−0.7	3.3	0.691	0.08
Weight (kg) ^a^	51.3 ± 2.6	25.2	118.2	54.6 ± 2.0	35.0	90.0	0.062	0.20
BMI (kg/m^2^) ^a^	25.5 ± 0.6	18.2	39.1	26.6 ± 0.5	19.5	36.0	0.085	0.25
BMI z-score ^a^	2.8 ± 0.1	1.5	4.3	3.4 ± 0.2	1.9	6.8	0.002	0.70
Overweight	3 (5.5)			2 (4.4)				
Obese	52 (94.6)			44 (95.7)				
WC (cm)	85.0 ± 1.7	62.0	121.0	90.1 ± 1.5	71.5	116.5	0.028	0.44
WC z-score	2.1 ± 0.0	1.4	3.1	2.1 ± 0.1	1.2	2.6	0.194	0.29
Waist:height	0.61 ± 0.01	0.50	0.71	0.63 ± 0.01	0.53	0.75	0.010	0.52
%BF	38.4 ± 0.6	29.0	48.3	36.3 ± 0.7	22.2	48.7	0.025	0.46
FM (kg) ^a^	20.0 ± 1.1	8.4	49.4	20.1 ± 1.0	9.1	41.4	0.551	0.02
FMI (kg/m^2^) ^a^	9.9 ± 0.3	5.5	16.5	9.8 ± 0.4	5.0	17.1	0.959	0.05
Trunk:limb FM	0.65 ± 0.01	0.41	0.90	0.66 ± 0.02	0.46	1.00	0.863	0.03
Android:gynoid	0.98 ± 0.01	0.80	1.21	1.01 ± 0.02	0.70	1.18	0.145	0.29
Android fat patterning	26 (47)			25 (54)				
Gynoid fat patterning	29 (53)			21 (46)				
LBM (kg) ^a,b^	30.0 ± 1.4	15.7	65	33.2 ± 1.1	22.9	57.3	0.018	0.34
LMI (kg/m^2^)	15.0 ± 0.3	11.4	22.0	16.1 ± 0.2	13.4	20.0	0.003	0.57
25(OH)D (nmol/L) ^a^	62.2 ± 2.4	30.7	108.0	62.2 ± 2.6	34.5	105.0	0.846	0.00
≥50 nmol/L	40 (72.7)			31 (67.4)				
30–49 nmol/L	15 (27.3))			15 (32.3)				
<30 nmol/L	0			0				
Vitamin D intake (IU/d) ^a^	175.9 ± 13.5	13.0	477.9	200.7 ± 20.4	7.7	741.6	0.399	0.21
≥EAR	2 (3.6)			2 (4.3)				
≥RDA	0 (0)			1 (2.2)				
Supplements used	6 (10.9)			1 (2.2)				
Sun index (min/d) ^a^	8.3 ± 2.4	0.0	112.4	13.7 ± 4.0	0.0	168.0	0.013	0.25
Sunscreen used	22 (40.0)			18 (39.1)				
UVB exposure period	23 (41.8)			31 (57.4)				
Ethnicity								
White	41 (74.6)			31 (67.4)				
Black	6 (10.9)			1 (2.2)				
Hispanic	2 (3.6)			1 (2.2)				
Mixed	4 (7.3)			10 (21.7)				
Asian	0			2 (4.4)				
Other	2 (3.6)			1 (2.2)				
Fitzpatrick skin type								
I-III	42 (77.8)			35 (76.1)				
IV-VI	12 (22.2)			11 (23.9)				
Pubertal status								
Tanner 1–3	50 (90.9)			43 (93.5)				
Tanner 4–5	5 (9.1)			3 (6.5)				
Mothers’ education level								
High school/college *	27 (51.9)			22 (48.9)				
University	24 (46.2)			23 (51.1)				
Refused to answer	1 (1.9)			0				
Family income								
< CAD 75,000	17 (32.1)			17 (37.8)				
≥ CAD 75,000	30 (56.6)			25 (55.6)				
Declined to answer	6 (11.3)			3 (6.7)				

Student’s *t*-test, mean ± SD reported, significance set at *p* < 0.05. Cohen’s *d* used to report effect size. For categorical data, *n*(%) reported. 25(OH)D = 25-hydroxyvitamin D; BF = body fat; BMI = body mass index; ES = effect size; FM = fat mass; FMI = fat mass index; LBM = lean body mass; WC = waist circumference. ^a^ Non-Gaussian distribution; Wilcoxon–Mann–Whitney U-test performed. ^b^ Excludes bone mass. * Includes technical and trade schools.

**Table 2:** Change in z-score between baseline and 12 mo in those who lost, maintained, and gained in each adiposity indicator.

	Loss				Maintenance				Gain				
Z-Score	*n*	Baseline	12-mo	*p*_time_	*N*	Baseline	12-mo	*p*_time_	*n*	Baseline	12-mo	*p*_time_	ES(*f*)
BMI	26	3.6 ± 0.2 ^a^	2.7 ± 0.2	<0.001	47	3.0 ± 0.1	2.8 ± 0.1	<0.001	28	2.7 ± 0.2 ^a^	2.9 ± 0.2	0.01	1.56
FMI	17	1.8 ± 0.1	1.0 ± 0.1 ^a,b^	<0.001	62	1.7 ± 0.1	1.5 ± 1.1 ^a^	<0.001	22	1.5 ± 0.1	1.7 ± 0.1 ^b^	<0.001	1.70
%BF	15	0.9 ± 0.1	0.1 ± 0.1 ^a,b^	<0.001	58	1.1 ± 0.1	0.9 ± 0.1 ^a^	<0.001	28	0.8 ± 0.1	1.0 ± 0.1 ^b^	<0.001	1.69
WC	8	2.0 ± 0.1	1.4 ± 0.1 ^a,b^	<0.001	70	2.2 ± 0.0 ^c^	2.0 ± 0.0 ^b^	<0.001	23	1.9 ± 0.1 ^c^	2.0 ± 0.1 ^a^	<0.001	1.57
Waist:height	10	1.8 ± 0.1	1.1 ± 0.1 ^a,b^	<0.001	60	2.0 ± 0.0	1.9 ± 0.0 ^a^	<0.001	31	1.8 ± 0.1	2.0 ± 0.1 ^b^	<0.001	1.59
Trunk:limb	31	0.3 ± 0.1 ^a^	−0.5 ± 0.1 ^b^	<0.001	45	0.0 ± 0.1	−0.2 ± 0.1	<0.001	25	−0.2 ± 0.1 ^a^	0.1 ± 0.1 ^b^	<0.001	1.92
Android:gynoid †	19	1.0 ± 0.0	0.9 ± 0.0 ^a,b^	<0.001	40	1.0 ± 0.0	1.0 ± 0.0 ^b^	0.30	42	1.0 ± 0.0	1.0 ± 0.0 ^a^	<0.001	2.10
LMI	29	3.4 ± 0.2	2.6 ± 0.2	<0.001	42	3.1 ± 0.1	2.9 ± 0.1	<0.001	30	2.9 ± 0.2	3.2 ± 0.2	<0.001	2.07

Mixed effects model, mean ± SEM. Same superscript signifies significant difference between loss, maintenance, and gain groups, *p* < 0.05. Effect size (ES) reported as Cohen’s *f*. † Android:gynoid not categorized by z-score; android–gynoid ratio ≥1 = android, android–gynoid ratio <1 = gynoid.

**Table 3:** Plasma 25(OH)D between baseline and 12 mo by category of change in adiposity z-score.

	25(OH)D (nmol/L)									
	Decrease			Maintenance			Increase			ES(*f*)
Z-Score	*n*	Baseline	12-mo	*n*	Baseline	12-mo	*n*	Baseline	12-mo	
BMI	26	57.1 ± 1.1	59.5 ± 1.1	47	55.5 ± 1.0	55.4 ± 1.0	28	57.2 ± 1.1	56.1 ± 1.1	0.15
FMI	17	62.7 ± 1.1	64.0 ± 1.1	62	56.1 ± 1.0	56.1 ± 1.0	22	53.4 ± 1.1	54.3 ± 1.1	0.14
%BF	15	63.0 ± 1.1	65.8 ± 1.1 *	58	57.7 ± 1.0	56.8 ± 1.0	28	52.2 ± 1.1	53.1 ± 1.1 *	0.14
WC	8	63.9 ± 1.1	64.6 ± 1.1	70	55.2 ± 1.0	57.1 ± 1.0	23	57.1 ± 1.1	53.4 ± 1.1	0.21
Waist:height	10	63.3 ± 1.1	65.6 ± 1.1	60	54.4 ± 1.0	55.4 ± 1.0	31	58.7 ± 1.1	56.8 ± 1.1	0.16
Trunk:limb	31	55.4 ± 1.1	58.6 ± 1.1	45	56.3 ± 1.0	56.2 ± 1.1	25	58.3 ± 1.1	55.3 ± 1.1	0.27
Android:gynoid †	19	54.4 ± 1.1	59.6 ± 1.1	40	52.1 ± 1.0	55.8 ± 1.1	42	57.3 ± 1.0	56.4 ± 1.0	0.22
LMI	29	55.3 ± 1.1	57.4 ± 1.1	42	56.2 ± 1.0	56.8 ± 1.0	30	57.3 ± 1.1	55.5 ± 1.1	0.18

Mixed effects model, mean ± SEM. Children categorized as having either decreased, maintained, or increased in each adiposity indicator at 12 mo compared to baseline. Covariates included sex, age, UVB exposure period, constitutive skin color, sunscreen use, dietary vitamin D intake; *p* < 0.05. Effect size (ES) reported as Cohen’s *f*. * Significant difference between %BF decrease and increase, no time effect. † Android:gynoid not categorized by z-score; android–gynoid ratio ≥1 = android, android–gynoid ratio <1 = gynoid.

## Data Availability

The data described in the manuscript will not be made available because permission to share data was not requested at the time of obtaining participant consent.

## Supplementary Materials

The following supporting information can be downloaded at: https://www.mdpi.com/article/10.3390/nu14153153/s1, Figure S1: Flow of participants; exclusions from final analysis; Table S2: Characteristics of participants by tertile of adiposity indicator at baseline.
