# Supplementary material for: Associations between Body Composition and Vitamin D Status in Children with Overweight and Obesity Participating in a 1-Year Lifestyle Intervention
Source: Nutrients. 2022 Jul 30;14(15):3153. doi: 10.3390/nu14153153 (PMC9370728; doi:10.3390/nu14153153)

Supplemental Table S1: Characteristics of participants by tertile of adiposity indicator at baseline

		Tertile 1 (n=34)	Tertile 2 (n=33)	Tertile 3 (n=34)
BMI (kg/m²)	Mean	23.1 ± 2.6	26.0 ± 3.0	28.8 ± 4.4
	Mean z-score	2.3 ± 0.3	2.9 ± 0.2	4.0 ± 0.9
	Age	9.6 ± 2.2	9.5 ± 1.7	8.8 ± 1.8
	% Male	30.3	47.1	58.8
BF (%)	Mean	33.3 ± 3.3	37.5 ± 2.5	41.5 ± 3.7
	Mean z-score	0.4 ± 0.4	1.0 ± 0.1	1.6 ± 0.3
	Age	9.3 ± 2.0	9.9 ± 2.0	8.7 ± 1.7
	% Male	32.4	51.5	52.9
FMI (kg/m²)	Mean	8.1 ± 1.5	9.7 ± 1.7	11.7 ± 2.7
	Mean z-score	1.2 ± 0.3	1.7 ± 0.1	2.2 ± 0.2
	Age	9.8 ± 2.0	9.5 ± 1.8	8.6 ± 1.8
	% Male	41.2	48.5	47.1
Adjusted FMI (kg/m²)	Mean	6.6 ± 1.0	8.3 ± 1.0	9.9 ± 2.0
	Mean z-score	1.2 ± 0.3	1.7 ± 0.1	2.2 ± 0.3
	Age	9.6 ± 2.1	9.8 ± 1.8	8.5 ± 1.7
	% Male	41.2	48.5	47.1
WC (cm)	Mean	81.1 ± 10.5	87.2 ± 9.9	93.6 ± 11.6
	Mean z-score	1.7 ± 0.2	2.1 ± 0.1	2.5 ± 0.2
	Age	9.7 ± 2.1	9.4 ± 1.8	8.8 ± 1.8
	% Male	44.1	39.4	52.9
Waist:height	Mean	0.57 ± 0.03	0.62 ± 0.02	0.67 ± 0.04
	Mean z-score	1.6 ± 0.3	1.9 ± 0.1	2.3 ± 0.2
	Age	9.2 ± 2.1	9.6 ± 1.9	9.1 ± 1.8
	% Male	35.3	51.5	50.0
Trunk:limb FM	Mean	0.54 ± 0.05	0.66 ± 0.05	0.76 ± 0.09
	Mean z-score	-0.7 ± 0.4	0.1 ± 0.2	0.8 ± 0.4
	Age	9.5 ± 1.9	9.5 ± 1.9	9.0 ± 1.7
	% Male	38.2	39.4	58.8
LMI (kg/m²)	Mean	13.9 ± 1.6	15.6 ± 1.4	16.9 ± 1.9
	Mean z-score	2.1 ± 0.4	3.1 ± 0.2	4.2 ± 0.5
	Age	9.3 ± 2.1	9.6 ± 1.7	9.0 ± 2.0
	% Male	35.3	54.6	47.1
Adjusted LMI (kg/m²)	Mean	9.9 ± 0.5	11.1 ± 0.4	12.0 ± 0.7
	Mean z-score	1.8 ± 0.4	2.9 ± 0.2	4.1 ± 0.7
	Age	9.2 ± 2.0	9.0 ± 2.0	9.7 ± 1.8
	%Male	38.2	51.5	47.1
		Android (n=51)	Gynoid (n=50)	
Android:Gynoid	Mean	1.1 ± 0.1	0.9 ± 0.1	
	Age	9.9 ± 1.8	8.7 ± 1.8	
	%Male	49.0	42.0	

Abbreviations: BMI=body mass index; BF=body fat; FMI=fat mass index; LMI=lean mass index; WC=waist circumference

Supplement Figure S1: Flow of participants; exclusions from final analysis

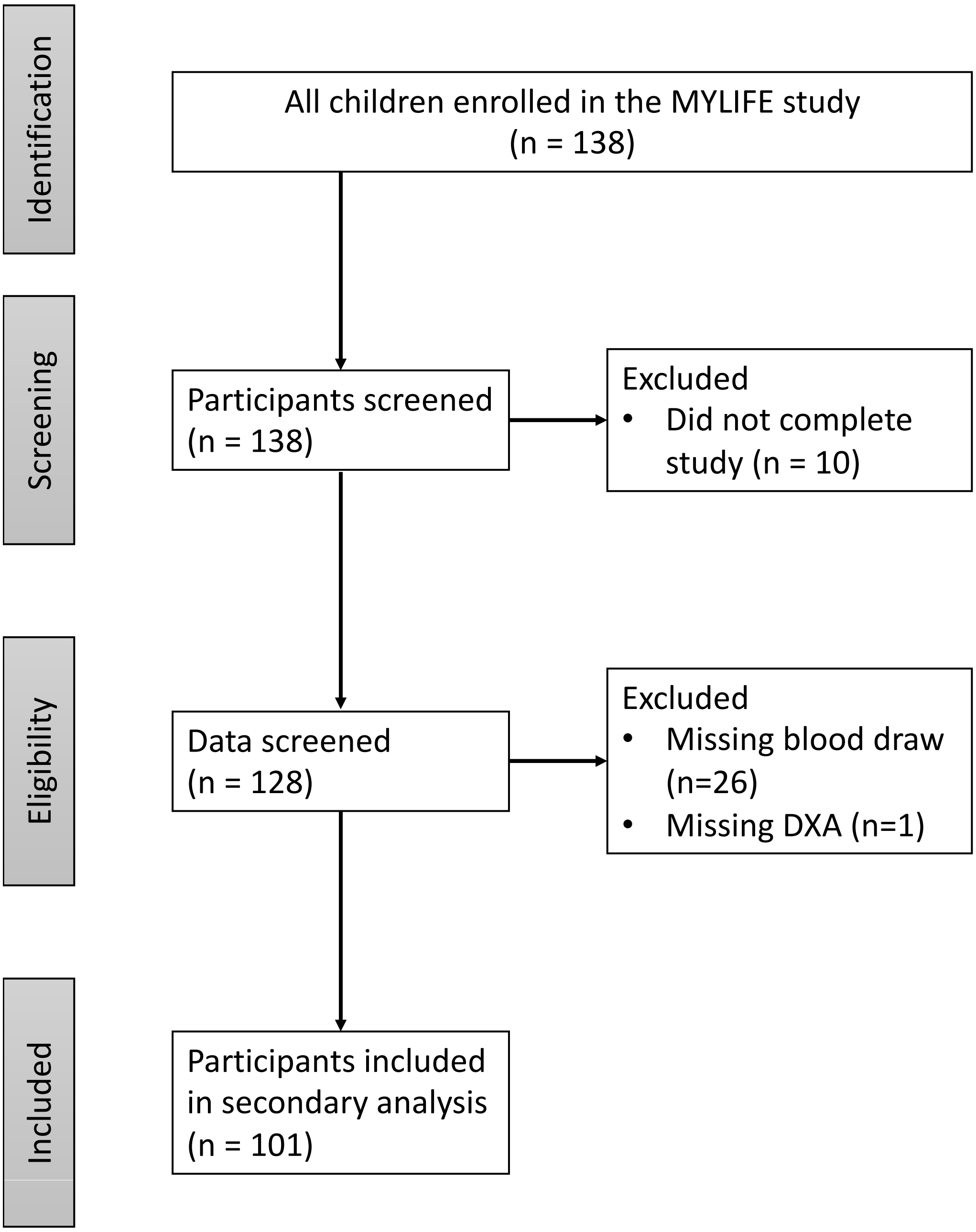

Supplement: Supplementary file 1 [file nutrients-14-03153-s001.zip › nutrients-1784549-supplementary.pdf]
